# Association between clinical evaluation and self perception of deglutition with motor disability scale in patients with multiple sclerosis

**DOI:** 10.1590/2317-1782/20212021026

**Published:** 2022-01-10

**Authors:** Dandara Tailuma Weiler Piloti, Vânia Carolina Devitte Ruiz, Marlise de Castro Ribeiro, Sheila Tamanini de Almeida

**Affiliations:** 1 Curso de Fonoaudiologia, Universidade Federal de Ciências da Saúde de Porto Alegre – UFCSPA - Porto Alegre (RS), Brasil.; 2 Departamento de Clínica Médica, Universidade Federal de Ciências da Saúde de Porto Alegre – UFCSPA - Porto Alegre (RS), Brasil.; 3 Departamento de Fonoaudiologia, Universidade Federal de Ciências da Saúde de Porto Alegre – UFCSPA - Porto Alegre (RS), Brasil.

**Keywords:** Multiple Sclerosis, Deglutition Disorders, Deglutition, Disability Evaluation, Adult

## Abstract

**Purpose:**

To investigate the association between the clinical evaluation and self-perception of deglutition with the motor disability scale in patients with Multiple Sclerosis.

**Methods:**

It is a cross-sectional, prospective study that was conducted with individuals with Multiple Sclerosis treated by the Neuroimmunology outpatient clinic of a hospital in southern Brazil. We reviewed the electronic medical records of patients to extract the score from the last Expanded Disability Status Scale. After the analysis of the inclusion criteria, and in clinical consultation, two protocols were applied: one of self-perception for the risk of dysphagia, through the Brazilian equivalence instrument of the Eating Assessment Tool; and the clinical evaluation of swallowing, with food, through the scale Gugging Swallowing Screen. The data were analyzed through tables, descriptive statistics and the tests: Fisher's Exact Association Test and Chi-square Test to assess the association between the results of the applied scales. We considered a maximum significance level of 5% (p <0.05).

**Results:**

It was possible to observe that there was a significant association between the scores of the Gugging Swallowing Screen scales with the Expanded Disability Status Scale of the patients. In addition, there was also a relation between the results of both protocols with the Expanded Disability Status Scale.

**Conclusion:**

The patients with Multiple Sclerosis in this study presented oropharyngeal dysphagia, what was confirmed by the association between the clinical evaluation of swallowing and the results of the instrument of self-perception of swallowing and the motor disability scale.

## INTRODUCTION

Demyelinating diseases constitute a group of pathologies in which damage to the myelin sheath occurs generally by affecting different areas of the central nervous system (CNS) ^(1)^. Within this group, there is Multiple Sclerosis (MS) which is a chronic neurological disease in which the individual's immune system degrades the myelin sheath causing damage - resulting from immune-mediated inflammation, demyelination and consequent axonal damage, neuronal loss and gliosis that lead to impairment of sensory and motor functions ^(2)^. The etiology of MS is not completely known, but it is believed to be multifactorial, with the influence of genetic and environmental factors contributing to the onset of the disease ^(3)^.

From an epidemiological point of view, MS generally affects Caucasians, young adults, from the second decade of life, and it is twice as frequent in women as in men. The estimated number of people with MS worldwide increased from 2.1 million in 2008 to 2.3 million in 2013 ^(4,5)^. The prevalence of MS found in studies carried out in Brazil ranged from 1.36/100,000 inhabitants in Recife (northwest region) to 27.2/100,000 inhabitants in the city of Santa Maria, Rio Grande do Sul (southern region) ^(6)^.

There are three distinct forms of MS manifestation, namely: relapsing-remitting, which is characterized by outbreaks of symptoms followed by total or partial remission, being the most frequent form ^(7)^; primarily progressive, characterized by progressive and cumulative neurological damage since the onset of the disease, without periods of specific relapses; and, secondarily progressive, which consists of an association between the other two manifestations, where after a relapsing-remitting period, the disease enters a phase in which there is clinical deterioration ^(7,8)^.

Pharmacological treatment aims to prevent the progression of the disease and modify its natural history. Until 2017, the Clinical Protocol and Therapeutic Guidelines for Multiple Sclerosis (CPTG) subdivided the drugs into first (beta-interferons and glatiramer), second (fingolimod and natalizumab) and third lines (fingolimod, in natalizumab failure). As of 2018, a new CPTG was created, which added teriflunomide in the first line and dimethyl fumarate in the second line, in addition to moving natalizumab to the third line in the treatment of MS ^(9)^.

MS is characterized by a variable combination of neurological symptoms such as: chronic pain, fatigue, visual, sensory, motor, coordination, balance, speech and/or swallowing changes, sphincter dysfunction and depression. Deficits caused by disease progression can undergo total or partial remission and, over time, all patients tend to show progressive neurological restrictions ^(3)^.

Oropharyngeal dysphagia is a symptom that can be identified in MS, which is characterized by the difficulty of swallowing in carrying the bolus from the oral cavity to the stomach. This disorder often results in damage to the individual's nutritional, pulmonary, food and social pleasure aspects ^(10)^. It is estimated that the incidence of dysphagia in patients with MS varies from 33 to 43%, and that it occurs more frequently in individuals who are at a more advanced stage, but it also manifests itself in patients with a lower level of impairment. In these patients, dysphagia can manifest as oral dysfunctions, fatigue during feeding, presence of stasis, penetration and/or aspiration ^(11)^. According to the literature, it is possible to observe the appearance of dysphagia in individuals with mild impairment on the Expanded Disability Status Scale (score <4) and the symptom intensifies when there is a greater impairment according to the scale, as there is an increase in disabilities functional, also affecting the dynamics of swallowing, but there are still few studies that correlate the EDSS scale score with the onset of oropharyngeal dysphagia ^(11,12)^.

In this context, the aim of this study is to investigate the association between clinical assessment and self-perceived swallowing with the motor disability scale in patients with Multiple Sclerosis.

## METHODS

The present research was approved by the Ethics and Research Committee of the proposing health institution under opinion number 2.021.922. This is a prospective cross-sectional study carried out through the collection of clinical data from patients diagnosed with Multiple Sclerosis treated at the Neuro-immunology clinic of a hospital in southern Brazil.

The inclusion criteria for this study were:

- Being over 18 years-old;
- Multiple Sclerosis Diagnosis;
- Being monitored and treated by the Neuro-immunology Service;
- Using first or second-line medication for the treatment of Multiple Sclerosis, in accordance with the Ministry of Health ordinance in force until the year 2017 ^(9)^.
- Agreement and assignment of the Informed Consent Form;

The exclusion criteria were:

- Another disease associated with Multiple Sclerosis;
- A contraindication for oral feeding;
- Using third-line medications for the treatment of Multiple Sclerosis in accordance with the Ministry of Health ordinance in force until the year 2017 ^(9)^.

Initially, the sample consisted of 51 patients diagnosed with MS evaluated between April 2017 and August 2018. After verifying the inclusion and exclusion criteria, three of these individuals were removed from the sample, two for not agreeing with the Informed Consent Form and one for having a discarded diagnosis of MS, totaling 48 study participants. The average age was 43 years-old, and out of these 34 (70.8%) are female. They presented disease diagnosis time of approximately nine years of disease course (Table 1). It was also found that the most frequent form of MS manifestation was relapsing-remitting with 37 individuals (77.1%), and that 26 (54.2%) of the patients in this sample were using first-line medication such as form of treatment.

**Table 1:** Description of study variables for sample characterization

Variable	Category	Nº of cases	%
Age	Up to 20	1	2.1
	21 - 40	21	43.8
	41 - 60	25	52.1
	Over 60	1	2.1
Gender	Female	34	70.8
	Male	14	29.2
Treatment	First Line Medicine	26	54.2
	Second Line Medicine	22	45.8
GUSS Result	Normal Swallowing	18	37.5
	Mild Dysphagia with Risk of Aspiration	29	60.4
	Moderate Dysphagia with Risk of Aspiration	1	2.1
EAT 10	Up to 3	27	56.3
	Over 3	21	43.8
EDSS	0 - 4	27	56.3
	Over 4	21	43.8
Type of MS	Relapsing-Remitting	37	77.1
	Primarily progressive	10	20.8
	Secondarily progressive	1	2.1
Time of diagnosis	0 - 5 years	20	41.7
	6 - 10 years	16	33.3
	11 - 15 years	5	10.4
	Over 15 years	7	14.6

GUSS - Gugging Swallowing Screen; EAT - Eating Assessment Tool; EDSS - Expanded Disability Status Scale; MS - Multiple Sclerosis.

First, a review of the electronic medical records of the patients and the appointment schedule of the Neuro-immunology Service was carried out. Thus, data such as age, sex, diagnosis, MRI result, time of diagnosis, proposed treatment (medication), comorbidities and score of the last Expanded Disability Status Scale (EDSS) were collected, this data was updated according to the consultation of the patient with the Neuro-immunology service, that is, the vast majority of patients were evaluated on the same day and some varied in a maximum of 60 days until they were submitted to direct swallowing assessment.

After this first phase of identification, patients from the Neuro-immunology Service who met the inclusion criteria were invited to participate in an interview and in a speech therapy assessment at the clinic. Initially, the Informed Consent Form was presented for the use of data collected exclusively for the research, and based on its agreement, the research continued.

The researcher applied the Brazilian equivalence protocol of Eating Assessment Tool (EAT-10) to later compare the reported complaints with the results of the clinical evaluation of swallowing. After filling out the EAT-10, the clinical evaluation of swallowing was performed using the Gugging Swallowing Screen (GUSS) scale ^(13)^.

The Expanded Disability Status Scale is a method to quantify disabilities that occur during the evolution of Multiple Sclerosis over time and is also a way to monitor changes in the levels of these disabilities. It has twenty items with scores ranging from zero to 10, with a score that increases by half a point according to the degree of impairment of the patient, with the cutoff: score > four showing clinical worsening of the disease ^(9,14)^.

The instrument of Brazilian equivalence of the EAT-10 is a self-assessment to identify the risk of dysphagia. This protocol consists of 10 simple questions, and provides information about functionality, emotional impact and physical symptoms that a problem with swallowing can cause in an individual's life. Each item is rated on a scale from zero (no problem) to four (severe problem). The total score is the sum of all answers, ranging from zero to 40 points. The cutoff point defined as the limit between “pass” and “failure” in feeding screening is three, with a higher risk for oropharyngeal dysphagia being above this value ^(15,16)^.

Consisting of seven items, the GUSS scale aims to assess the capacity and degree of swallowing problems, as well as to identify adequate guidance on nutrition and/or additional investigations. The results predicted by this scale classify swallowing as: normal, mild dysphagia, moderate dysphagia or severe dysphagia ^(13)^.

The evaluations consisted of the offer of the three food consistencies provided for by GUSS, namely: pasty (grape juice thickened in pudding consistency with industrial thickener), liquid (water) and solid (loaf bread), accompanied by cervical auscultation with the aid of stethoscope (Litmann® Classic II Pediatric) for the evaluation of signs suggestive of laryngeal penetration and/or tracheal aspiration. After completing the protocols, the results were discussed with the medical team responsible for the cases, and presented to the patients and their caregivers, with guidance on the signs and symptoms of dysphagia, and when it was necessary, the appropriate referrals to speech therapy were carried out or for additional exams.

At the end of collection, all data were organized in a spreadsheet on Excel software version 16.0 and analyzed through tables, descriptive statistics and statistical tests: Fisher's Exact Association Test and Chi-square test to assess the association between the results of the scales applied. The results were considered significant at a maximum significance level of 5% (p<0.05) and the software used for this analysis was SPSS version 22.0.

## RESULTS

It was found that most patients (56.3%) had a score on the EDSS scale of up to four points, evidencing a mild motor impairment (Table 1). Through the results of the Chi-square and Fisher's exact association tests, it was found that the treatment variables, time since diagnosis, EAT-10 and GUSS scores were significantly associated with the patients' EDSS score. Individuals treated with first-line drugs had less impairment of functional capacities than those treated with second-line drugs, this is perhaps explained by the choice of treatment according to the course of the disease.

The time since diagnosis of the disease between 11 and 15 years was associated with a greater impairment in the scale of motor disability - EDSS, as well as the results of the EAT-10 and GUSS demonstrate that they are directly proportional to the EDSS. That is, the lower the EDSS the lower the EAT-10 score, and the lower the chances of patients presenting dysphagia according to the clinical assessment of swallowing (Table 2).

**Table 2:** Comparisons of variables with EDSS scale results

Variable	Category	EDSS				p
		0 - 4		Over 4		
		n	%	n	%	
Treatament^1^	First line medicine	**19**	**70.4**	7	33.3	0.019^a^
	Second line medicine	8	29.6	**14**	**66.7**	
Time of diagnosis^2^	0 - 5 years	13	48.1	7	33.3	0.000^b^
	6 - 10 years	9	33.3	7	33.3	
	11 - 15 years	-	-	**5**	**23.8**	
	Over 15 years	5	18.5	2	9.5	
Type of MS^2^	Relapsing-remitting	26	96.3	11	52.4	0.051^c^
	Primarily progressive	1	3.7	9	42.9	
	Secondarily progressive	-	-	1	4.8	
EAT 10^1^	Up to 3	**19**	**70.4**	8	38.1	0.040^a^
	Over 3	8	29.6	**13**	**61.9**	
GUSS Result^1^	Normal Swallowing	**14**	**51.9**	4	19.0	0.034^a^
	Mild Dysphagia with Risk of Aspiration	13	48.1	**16**	**76.2**	
	Moderate Dysphagia with Risk of Aspiration	-	-	1	4.8	

a Significant p≤0,05;

b Significant p≤0,01;

c Non-significant;

1 Chi-square test;

2 Fisher's exact test

GUSS - Gugging Swallowing Screen; EAT - Eating Assessment Tool; EDSS - Expanded Disability Status Scale; MS - Multiple Sclerosis.

In this sample, 29 (60.4%) of the patients had mild dysphagia (Table 3). Through the results of Fisher's Exact test, it was found that patients treated with first-line drugs had normal swallowing and the others had mild dysphagia. Likewise, the result of the EAT-10 with a score of up to three was related to normal swallowing and above that to oropharyngeal dysphagia.

**Table 3:** Comparisons of variables with GUSS scale results

Variable	Category	GUSS Resuly						p
		Normal Swallowing		Mild Dysphagia with Risk of Aspiration		Moderate Dysphagia with Risk of Aspiration		
		n	%	n	%	n	%	
Treatament¹	First line medicine	**17**	**94.4**	8	27.6	1	100.0	0.000^a^
	Second line medicine	1	5.6	**21**	**72.4**	-	-	
Type of MS¹	Relapsing-remitting	16	88.9	21	72.4	-	-	0.185^b^
	Primarily progressive	2	11.1	7	24.1	1	100.0	
	Secondarily progressive	-	-	1	3.4	-	-	
Time of diagnosis^1^	0 - 5 years	9	50.0	10	34.5	1	100.0	0.807^b^
	6 - 10 years	5	27.8	11	37.9	-	-	
	11 - 15 years	1	5.6	4	13.8	-	-	
	Over 15 years	3	16.7	4	13.8	-	-	
EAT 10^1^	Up to 3	**15**	**83.3**	11	37.9	1	100.0	0.003^a^
	Over 3	3	16.7	**18**	**62.1**	-	-	

a Significant p≤0,01;

b Non-significant;

1 Fisher's exact test

GUSS - Gugging Swallowing Screen; EAT - Eating Assessment Tool; MS - Multiple Sclerosis.

In Table 4, which refers to the EAT-10, it was identified that 27 (56.3%) patients had a score below three in relation to self-perceived risk of dysphagia. There was a relationship of two variables with the results of the EAT-10: the type of medication used in the treatment and the type of MS. Patients treated with first-line drugs have fewer complaints of swallowing disorders than those treated with second-line drugs, and patients with relapsing-remitting MS show less symptom perception when compared to patients with primarily progressive MS.

**Table 4:** Comparisons of variables with EAT-10 scale results

Variable	Category	EAT 10				p
		Up to 3		Over 3		
		n	%	n	%	
Treatament^1^	First line medicine	**20**	**74.1**	6	28.6	0.003^b^
	Second line medicine	7	25.9	**15**	**71.4**	
Type of MS^2^	Relapsing-remitting	**24**	**88.9**	13	61.9	0.048^a^
	Primarily progressive	3	11.1	**7**	**33.3**	
	Secondarily progressive	-	-	**1**	**4.8**	
Time of diagnosis	0 - 5 years	10	37.0	10	47.6	0.361^c^
	6 - 10 years	9	33.3	7	33.3	
	11 - 15 years	2	7.4	3	14.3	
	Over 15 years	6	22.2	1	4.8	

a Significant p≤0,05;

b Significant p≤0,01;

c Non-significant;

1 Chi-square test;

2 Fisher's exact test

EAT - Eating Assessment Tool; MS - Multiple Sclerosis

## DISCUSSION

The profile of patients found in this sample is in line with the findings in the literature, which report that the number of patients diagnosed with MS was higher in females, as well as the most frequent type of MS was relapsing-remitting ^(2,3,8)^. Patients with MS have progressive neurological deficits, with changes in the motor and sensory systems, and with a potential functional impact on swallowing. Dysphagia is more related to the duration of the disease, as evidenced in this study, and can lead to several complications such as: bronchoaspiration pneumonia, dehydration and malnutrition, worsening the quality of life of these patients, and sometimes progressing to death ^(17,18)^.

It is known that the EDSS is an instrument that quantifies the disabilities that occur during the evolution of MS over time and it is estimated that the longer the time since diagnosis of the disease, the higher its score, as identified in this research ^(14)^. It is also understood that dysphagia is more common in patients with more severe conditions of the disease, although the EDSS scale scores demonstrate that some of the individuals in this sample had mild impairment in swallowing with EDSS scores up to four ^(19)^. However, it is expected that with the progression of MS patients present even greater risks for dysphagia due to their motor and/or sensory impairment ^(20)^. Tarameshlu et al. identified in their study that patients with dysphagia had significantly higher EDSS scores than non-dysphagic patients. Furthermore, they also observed that the duration of the disease was significantly longer in the group of dysphagic patients ^(12)^.

First-line drugs are used in patients with early-stage MS and less active disease, that is, with fewer functional disabilities. Second and third-line medications are used in individuals with more aggressive disease progression, and who have already failed with the first therapeutic strategy ^(9,19)^. Although there are not enough subsidies to indicate that the type of drug has an influence on dysphagia, it was observed that the evolution of the disease in relation to the time of diagnosis and the EDSS scale scores above the cutoff point showed a relationship with the change. of swallowing.

In this sample, the number of patients with MS who reported symptoms suggestive of swallowing disorders was significant, considering their self-perception through the application of the EAT-10 questionnaire. It indicated that there is interference in the results by the treatment and also by the form of presentation of the disease, suggesting that patients medicated with a first-line drug would have fewer complaints of swallowing, due to the initial stage of the disease and, consequently, lower EDSS. Likewise, individuals with relapsing-remitting MS had fewer complaints than patients with primarily progressive MS.

There are other self-perception protocols adopted to identify dysphagia in patients with neurodegenerative diseases, in particular, the DYMUS for people with Multiple Sclerosis. However, this is an instrument that is limited to questions related to the act of swallowing, and not to the social and psychological impacts of these individuals caused by a swallowing disorder ^(13,16)^. The EAT-10 is an useful instrument to detect the existence of dysphagia and monitor an individual's response to treatment ^(13)^. This questionnaire proved to be useful as a means of screening for dysphagia, as almost half of the patients with MS in this sample reported signs suggestive of swallowing disorders as a result of the questionnaire, which is associated with the level of dysphagia identified by the GUSS. In a previous study, the presence of oropharyngeal dysphagia was identified in 90% of patients evaluated with MS. Dysphagia severity was related to the clinical form of the disease, and it was found that severe dysphagia was more present in the primarily progressive and secondarily progressive forms, and mild and moderate dysphagia was more found in the relapsing-remitting form ^(21)^. These data corroborate the results of the GUSS and EAT-10 scales of this study, which show that more than half of the individuals in this sample have mild oropharyngeal dysphagia, as well as relapsing-remitting MS.

The study had limitations to assert about the interference of the proposed treatment in relation to the risk of dysphagia, due to the heterogeneity of the groups and the different motor impairments of the patients evidenced by the EDSS scale. The other results are consistent with the findings in the literature, and it is possible to state that the self-perception of the risk of dysphagia in individuals with MS demonstrated by the EAT-10 is related to the findings of the clinical evaluation of swallowing carried out using the GUSS. Further research with homogeneous groups is also suggested for more reliable results in relation to the type of MS, the severity of dysphagia and the treatment of MS and its influence on swallowing disorders.

## CONCLUSION

It can be said that there was an association between the findings of the clinical evaluation, the self-perception of swallowing instrument and the scale of motor disability in patients with MS.
