# Supplementary material for: Association between clinical evaluation and self perception of deglutition with motor disability scale in patients with multiple sclerosis
Source: Codas. 2022 Jan 10;34(2):e20210026. doi: 10.1590/2317-1782/20212021026 (PMC9769428; doi:10.1590/2317-1782/20212021026)
Supplement: Translation

# Translation: Associação entre avaliação clínica e autopercepção da deglutição com a escala de incapacidade motora em pacientes com esclerose múltipla

**Authors:** Dandara Tailuma Weiler Piloti, Vânia Carolina Devitte Ruiz, Marlise de Castro Ribeiro, Sheila Tamanini de Almeida

**Affiliations:** 1 Curso de Fonoaudiologia, Universidade Federal de Ciências da Saúde de Porto Alegre – UFCSPA - Porto Alegre (RS), Brasil.; 2 Departamento de Clínica Médica, Universidade Federal de Ciências da Saúde de Porto Alegre – UFCSPA - Porto Alegre (RS), Brasil.; 3 Departamento de Fonoaudiologia, Universidade Federal de Ciências da Saúde de Porto Alegre – UFCSPA - Porto Alegre (RS), Brasil.

**Keywords:** Esclerose Múltipla, Transtornos de Deglutição, Deglutição, Doenças Degenerativas, Adulto

## Abstract

**Objetivo:**

Investigar a associação entre a avaliação clínica e autopercepção da deglutição com a escala de incapacidade motora em pacientes com Esclerose Múltipla.

**Método:**

Estudo transversal, prospectivo realizado com indivíduos com Esclerose Múltipla atendidos pelo ambulatório de Neuroimunologia de um hospital do sul do Brasil. Realizamos a revisão dos prontuários eletrônicos dos pacientes para extração do escore da última Expanded Disability Status Scale. Após a análise dos critérios de inclusão, e em consulta clínica, foram aplicados dois protocolos, o de autopercepção para o risco de disfagia, através do instrumento de equivalência brasileira do Eating Assessment Tool e a avaliação clínica da deglutição, com alimentos, através da escala Gugging Swallowing Screen. Os dados foram analisados através de tabelas, estatísticas descritivas e pelos testes: Teste de Associação Exato de Fisher e Teste Qui-quadrado para avaliar a associação entre os resultados das escalas aplicadas. Consideramos um nível de significância máximo de 5% (p<0,05).

**Resultados:**

Foi possível observar que houve associação significativa entre os escores das escalas Gugging Swallowing Screen com a Expanded Disability Status Scale dos pacientes. Além disso, também se observou relação entre os resultados de ambos protocolos com a Expanded Disability Status Scale.

**Conclusão:**

Os pacientes com Esclerose Múltipla deste estudo apresentaram disfagia orofaríngea. Houve associação entre os achados da avaliação clínica, do instrumento de autopercepção da deglutição e da escala de incapacidade motora em pacientes com esclerose múltipla.

## INTRODUÇÃO

As doenças desmielinizantes constituem um grupo de patologias nas quais ocorre lesão da bainha de mielina, em geral afetando diferentes áreas do sistema nervoso central (SNC) ^(1)^. Dentro deste grupo, há a Esclerose Múltipla (EM) que se trata de uma doença neurológica crônica em que o sistema imunológico do indivíduo degrada a bainha de mielina causando lesões - resultantes de uma inflamação imunomediada, desmielinização e consequente, dano axonal, perda neuronal e gliose que levam a comprometimentos das funções sensitivas e motoras ^(2)^. A etiologia da EM ainda não é completamente conhecida, mas acredita-se que seja multifatorial com influência de fatores genéticos e ambientais contribuindo para o desencadeamento da doença ^(3)^.

Do ponto de vista epidemiológico, a EM geralmente acomete caucasianos, adultos jovens, a partir da segunda década de vida, e é duas vezes mais frequente em mulheres do que em homens. O número estimado de pessoas com EM no mundo aumentou de 2,1 milhões em 2008 para 2,3 milhões em 2013 ^(4,5)^. As prevalências de EM encontradas em estudos realizados no Brasil variaram de 1,36 / 100.000 habitantes em Recife (região noroeste) a 27,2 / 100.000 habitantes na cidade de Santa Maria, Rio Grande do Sul (região sul)^(6)^.

Existem três formas distintas de manifestação da EM sendo elas: remitente-recorrente ou surto-remissão, que caracteriza-se por surtos de sintomas seguidos de remissão total ou parcial deles, sendo a forma mais frequente ^(7)^; primariamente progressiva, caracterizada por lesão neurológica progressiva e cumulativa desde o início da doença, sem períodos de surtos específicos; e, secundariamente progressiva que consiste em uma associação entre as outras duas manifestações, onde após um período surto-remissão, a doença entra em uma fase em que há deterioração clínica ^(7,8)^.

O tratamento farmacológico visa impedir a progressão da doença e modificar a história natural dela. Até o ano de 2017, o Protocolo Clínico e Diretrizes Terapêuticas da Esclerose Múltipla (PCDT) subdividia as drogas em primeira (betainterferonas e glatirâmer), segunda (fingolimodes e natalizumabe) e terceira linhas (fingolimode, na falha de natalizumabe). A partir de 2018, foi criado um novo PCDT, que acrescentou a teriflunomida na primeira linha e fumarato de dimetila na segunda linha, além de passar o natalizumabe para terceira linha no tratamento da EM ^(9)^.

A EM caracteriza-se por uma combinação variável de sintomas neurológicos como: dor crônica, fadiga, alterações visuais, sensitivas, motoras, na coordenação, no equilíbrio, na fala e/ou na deglutição, disfunção esfincteriana e depressão. Os déficits ocasionados pela progressão da doença podem sofrer remissão total ou parcial e, com o decorrer do tempo, todos os pacientes tendem a mostrar restrições neurológicas progressivas ^(3)^.

A disfagia orofaríngea é um sintoma possível de ser identificado na EM e se caracteriza pela dificuldade de deglutição na condução do bolo alimentar da cavidade oral até o estômago. Este distúrbio frequentemente resulta em prejuízos aos aspectos nutricionais, pulmonar, prazer alimentar e social do indivíduo ^(10)^. Estima-se que a incidência de disfagia em pacientes com de EM varia de 33 a 43% e que ocorre com mais frequência em indivíduos que se encontram num estágio mais avançado, porém também pode se manifestar em pacientes com menor nível de comprometimento. Nestes pacientes a disfagia pode surtir com disfunções orais, fadiga durante a alimentação, presença de estase, penetração e/ou aspiração ^(11)^. De acordo com a literatura, pode-se observar o aparecimento da disfagia em indivíduos que apresentam comprometimento leve na *Expanded Desability Status Scale* (EDSS: escore <4) e como um sintoma intensificado quando há um comprometimento maior segundo a escala, já que existe um aumento das incapacidades funcionais, repercutindo também na dinâmica da deglutição ^(11)^.

Atualmente, poucos estudos correlacionam escalas motoras, como a EDSS, com a disfagia ou com seu grau de gravidade em pacientes com EM ^(11-12)^. Esta relação pode tornar-se uma aliada da equipe multiprofissional no processo de acompanhamento e prevenção de complicações pela evolução da doença. Neste contexto, o objetivo do presente estudo é investigar a associação entre a avaliação clínica, a avaliação de autopercepção da deglutição e a escala de incapacidade motora em pacientes com Esclerose Múltipla.

## MÉTODO

A presente pesquisa foi aprovada pelo Comitê de Ética e Pesquisa da instituição de saúde proponente através do parecer de número 2.021.922. Trata-se de um estudo transversal prospectivo realizado a partir da coleta de dados clínicos de pacientes com diagnóstico de Esclerose Múltipla atendidos no ambulatório de Neuroimunologia de um hospital do sul do Brasil.

Os critérios de inclusão para esse estudo foram:

- Idade acima de 18 anos;
- Diagnóstico de Esclerose Múltipla;
- Estar sendo acompanhado e tratado pelo Serviço de Neuroimunologia;
- Fazer uso de medicamento de primeira ou segunda linhas para tratamento de Esclerose Múltipla, de acordo com a portaria do Ministério da Saúde vigente até o ano de 2017 ^(9)^.
- Ter concordado e assinado o Termo de Consentimento Livre e Esclarecido;

Os critérios de exclusão foram:

- Ter outra doença associada a Esclerose Múltipla;
- Ter contraindicação de alimentação por via oral;
- Utilizar medicações de terceira linha para tratamento de Esclerose Múltipla, de acordo com a portaria do Ministério da Saúde vigente até o ano de 2017 ^(9)^.

Inicialmente a amostra foi composta por 51 pacientes diagnosticados com EM avaliados entre o período de abril de 2017 até agosto de 2018. Após verificar os critérios de inclusão e exclusão, três desses indivíduos foram retirados da amostra, dois por não concordarem com o Termo de Consentimento Livre e Esclarecido e um por ter diagnóstico de EM descartado, totalizando 48 participantes do estudo. A média de idade foi de 43 anos sendo que destes 34 (70,8%) eram do sexo feminino. Em relação ao tempo de diagnóstico da doença, observou uma média de aproximadamente nove anos de curso da doença (Tabela 1). Verificou-se também que a forma mais frequente de manifestação da EM foi a de surto-remissão com 37 indivíduos (77,1%), e que 26 (54,2%) dos pacientes dessa amostra faziam uso de medicação de primeira linha como forma de tratamento.

**Tabela 1:** Descrição das variáveis do estudo para a caracterização da amostra

Variável	Categoria	Nº casos	%
Idade	Até 20	1	2,1
	21 - 40	21	43,8
	41 - 60	25	52,1
	Mais de 60	1	2,1
Sexo	Feminino	34	70,8
	Masculino	14	29,2
Tratamento	Fármacos de Primeira Linha	26	54,2
	Fármacos de Segunda Linha	22	45,8
Resultado GUSS	Deglutição Normal	18	37,5
	Disfagia Leve com Risco de Aspiração	29	60,4
	Disfagia Moderada com Risco de Aspiração	1	2,1
EAT 10	Até 3	27	56,3
	Acima de 3	21	43,8
EDSS	0 - 4	27	56,3
	Acima de 4	21	43,8
Tipo de EM	Surto - Remissão	37	77,1
	Primariamente progressiva	10	20,8
	Secundariamente progressiva	1	2,1
Tempo de Diagnóstico	0 - 5 anos	20	41,7
	6 - 10 anos	16	33,3
	11 - 15 anos	5	10,4
	Acima de 15 anos	7	14,6

GUSS - Gugging Swallowing Screen; EAT - Eating Assessment Tool; EDSS - Expanded Disability Status Scale; EM - Esclerose Múltipla

Realizou-se, primeiramente, revisão dos prontuários eletrônicos dos pacientes e da agenda dos atendimentos do Serviço de Neuroimunologia. Dessa forma, foram coletados dados como idade, sexo, diagnóstico, resultado da RM, tempo de diagnóstico, tratamento proposto (medicação), comorbidades e o escore da *Expanded Disability Status Scale* (EDSS). Este dado era atualizado de acordo com a consulta do paciente no serviço de Neuroimulogia. A grande maioria dos pacientes foram avaliados no mesmo dia da avaliação direta de deglutição. Não havendo esta possibilidade, o escore utilizado era o da última consulta do mês de inclusão do paciente em nosso estudo.

Após esta primeira fase de identificação, os pacientes do Serviço de Neuroimunologia que se enquadraram nos critérios de inclusão, foram convidados a participar de uma entrevista e de uma avaliação fonoaudiológica no ambulatório. Foi apresentado, inicialmente, o Termo de Consentimento Livre e Esclarecido para utilização dos dados coletados exclusivamente para a pesquisa, e a partir da concordância do mesmo, prosseguia-se a pesquisa.

Foi aplicado, pela pesquisadora, o protocolo de equivalência brasileira do *Eating Assessment Tool* (EAT-10) para posterior comparação das queixas relatadas com os resultados da avaliação clínica da deglutição. Após o preenchimento da EAT-10, realizava-se a avaliação clínica da deglutição através da escala *Gugging Swallowing Screen* (GUSS) ^(13)^.

A *Expanded Disability Status Scale* trata-se de um método quantificador de incapacidades ocorridas durante a evolução da Esclerose Múltipla ao longo do tempo e também é uma forma de monitorar mudanças nos níveis destas incapacidades. Possui vinte itens com escores que variam de zero a 10, com pontuação que aumenta meio ponto conforme o grau de prejuízo do paciente tendo como corte: escore maior que quatro evidenciando piora clínica da doença ^(9,14)^.

Já o instrumento de equivalência brasileira do EAT-10 trata-se de uma autoavaliação de identificação do risco de disfagia. Esse protocolo é composto por 10 perguntas simples, e fornece informações sobre a funcionalidade, o impacto emocional e sintomas físicos que um problema de deglutição pode acarretar na vida de um indivíduo. Cada item é classificado em uma escala de zero (sem problema) a quatro (problema grave). A pontuação total é a soma de todas as respostas, variando de zero a 40 pontos. O ponto de corte definido como limite entre “passa” e “falha” na triagem da alimentação é de três, sendo acima desse valor, um risco maior para disfagia orofaríngea ^(15,16)^.

Composta por sete itens, a escala GUSS tem como objetivo avaliar a capacidade e grau dos problemas de deglutição, bem como identificar a orientação adequada sobre nutrição e/ou investigações adicionais. Os resultados previstos por essa escala, classificam a deglutição em: normal, disfagia leve, disfagia moderada ou disfagia grave ^(13)^.

As avaliações consistiam na oferta das três consistências alimentares previstas pela GUSS, sendo essas: pastosa (suco no sabor uva espessado na consistência pudim com espessante industrial), líquida (água) e sólida macia (pão de forma), acompanhadas da ausculta cervical com auxílio de estetoscópio (Litmann® Classic II Pediatric) para a avaliação de sinais sugestivos de penetração laríngea e/ou aspiração traqueal. Após a finalização do preenchimento dos protocolos, os resultados foram discutidos com a equipe médica responsável pelos casos e apresentados aos pacientes e seus acompanhantes, sendo realizadas orientações acerca dos sinais e sintomas de disfagia, e quando necessário, foram realizados os devidos encaminhamentos para terapia fonoaudiológica ou para exames complementares.

Ao final da coleta, todos os dados foram organizados em uma planilha através do software Excel versão 16.0 e analisados através de tabelas, estatísticas descritivas e pelos testes estatísticos: Teste de Associação Exato de Fisher e Teste Qui-quadrado para avaliar a associação entre os resultados das escalas aplicadas. Os resultados foram considerados significativos a um nível de significância máximo de 5% (p<0,05) e o software utilizado para esta análise foi o SPSS versão 22.0.

## RESULTADOS

Constatou-se a maioria dos pacientes (56,3%), apresentaram escore da escala EDSS de até quatro pontos, evidenciando um comprometimento motor leve (Tabela 1). Através dos resultados dos testes de associação Qui-quadrado e Exato de Fisher verificou-se que as variáveis de tratamento, tempo de diagnóstico, escore da EAT-10 e GUSS estiveram significativamente associadas ao escore do EDSS dos pacientes. Os indivíduos tratados com fármacos de primeira linha apresentaram menor comprometimento das capacidades funcionais do que os tratados com drogas de segunda linha, isso explica-se talvez pela escolha de tratamento de acordo com o curso da doença.

O tempo de diagnóstico da doença entre 11 e 15 anos esteve associado com um comprometimento maior na escala de incapacidade motora - EDSS, assim como os resultados da EAT-10 e da GUSS demonstram ser grandezas diretamente proporcionais a EDSS. Ou seja, quanto menor a EDSS menor o escore da EAT-10 e menores as possibilidades de os pacientes apresentarem disfagia de acordo com a avaliação clínica de deglutição (Tabela 2).

**Tabela 2:** Comparações das variáveis com os resultados da escala EDSS

Variável	Categoria	EDSS				p
		0 - 4		Acima de 4		
		n	%	n	%	
Tratamento^1^	Fármacos de Primeira Linha	**19**	**70,4**	7	33,3	0,019^a^
	Fármacos de Segunda Linha	8	29,6	**14**	**66,7**	
Tempo de Diagnóstico^2^	0 - 5 anos	13	48,1	7	33,3	0,000^b^
	6 - 10 anos	9	33,3	7	33,3	
	11 - 15 anos	-	-	**5**	**23,8**	
	Acima de 15 anos	5	18,5	2	9,5	
Tipo de EM^2^	Surto - Remissão	26	96,3	11	52,4	0,051^c^
	Primariamente progressiva	1	3,7	9	42,9	
	Secundariamente progressiva	-	-	1	4,8	
EAT 10^1^	Até 3	**19**	**70,4**	8	38,1	0,040^a^
	Acima de 3	8	29,6	**13**	**61,9**	
Resultado GUSS^1^	Deglutição Normal	**14**	**51,9**	4	19,0	0,034^a^
	Disfagia Leve com Risco de Aspiração	13	48,1	**16**	**76,2**	
	Disfagia Moderada com Risco de Aspiração	-	-	1	4,8	

a Significativo p≤0,05;

b Significativo p≤0,01;

c Não significativo;

1 Teste Qui-quadrado;

2 Teste Exato de Fisher

GUSS - Gugging Swallowing Screen; EAT - Eating Assessment Tool; EDSS - Expanded Disability Status Scale; EM - Esclerose Múltipla.

Nesta amostra, 29 (60,4%) dos pacientes apresentaram disfagia leve (Tabela 3). Através dos resultados do teste Exato de Fisher verificou-se que pacientes tratados com medicamentos de primeira linha apresentaram deglutição normal e os demais, disfagia leve. Do mesmo modo, que o resultado do EAT-10 com escore até três relacionou-se com deglutição normal e acima de disso à disfagia orofaríngea.

**Tabela 3:** Comparações das variáveis com os resultados da escala GUSS

Variável	Categoria	Resultado GUSS						p
		Deglutição Normal		Disfagia Leve com Risco de Aspiração		Disf. Mod. c/ Risco de Aspiração		
		n	%	n	%	n	%	
Tratamento1	Fármacos de Primeira Linha	**17**	**94,4**	8	27,6	1	100,0	0,000^a^
	Fármacos de Segunda Linha	1	5,6	**21**	**72,4**	-	-	
Tipo de EM^¹^	Surto - Remissão	16	88,9	21	72,4	-	-	0,185^b^
	Primariamente progressiva	2	11,1	7	24,1	1	100,0	
	Secundariamente progressiva	-	-	1	3,4	-	-	
Tempo de Diagnóstico¹	0 - 5 anos	9	50,0	10	34,5	1	100,0	0,807^b^
	6 - 10 anos	5	27,8	11	37,9	-	-	
	11 - 15 anos	1	5,6	4	13,8	-	-	
	Acima de 15 anos	3	16,7	4	13,8	-	-	
EAT 10¹	Até 3	**15**	**83,3**	11	37,9	1	100,0	0,003^a^
	Acima de 3	3	16,7	**18**	**62,1**	-	-	

a Significativo p≤0,01;

b Não significativo;

¹Teste Exato de Fisher

GUSS - Gugging Swallowing Screen; EAT - Eating Assessment Tool; EM - Esclerose Múltipla

Na Tabela 4, que se refere ao EAT-10, identificou-se que 27 (56,3%) pacientes apresentaram escore abaixo de três em relação a autopercepção do risco de disfagia. Verificou-se relação de duas variáveis com os resultados do EAT-10: o tipo de medicação utilizado no tratamento e o tipo de EM. Pacientes tratados com fármacos de primeira linha apresentam menos queixas de alterações de deglutição do que os tratados com medicamentos de segunda linha e pacientes com EM do tipo surto-remissão demonstram menor percepção de sintomas quando comparados com pacientes com EM do tipo primariamente progressiva.

**Tabela 4:** Comparações das variáveis com os resultados da escala EAT-10

Variável	Categoria	EAT 10				p
		Até 3		Acima de 3		
		n	%	n	%	
Tratamento^1^	Fármacos de Primeira Linha	**20**	**74,1**	6	28,6	0,003^b^
	Fármacos de Segunda Linha	7	25,9	**15**	**71,4**	
Tipo da EM^2^	Surto - Remissão	**24**	**88,9**	13	61,9	0,048^a^
	Primariamente progressiva	3	11,1	**7**	**33,3**	
	Secundariamente progressiva	-	-	**1**	**4,8**	
Tempo de diagnóstico	0 - 5 anos	10	37,0	10	47,6	0,361^c^
	6 - 10 anos	9	33,3	7	33,3	
	11 - 15 anos	2	7,4	3	14,3	
	Acima de 15 anos	6	22,2	1	4,8	

a Significativo p≤0,05;

b Significativo p≤0,01;

c Não significativo;

1 Teste Qui-quadrado;

2 Teste Exato de Fisher

EAT - Eating Assessment Tool; EM - Esclerose Múltipla

## DISCUSSÃO

O perfil de pacientes encontrados nesta amostra vai ao encontro dos achados da literatura, os quais referem que o número de pacientes com diagnóstico de EM mostrou-se maior em indivíduos do sexo feminino, assim como, o tipo de EM mais frequente foi de surto-remissão ^(2,3,8)^. Pacientes com EM apresentam déficits neurológicos progressivos, com modificações nos sistemas motores e sensitivos, e com potencial impacto funcional sobre a deglutição. A disfagia está mais relacionada ao tempo de duração da doença, como evidenciada no presente estudo, e pode levar a várias complicações como: pneumonia broncoaspirativa, desidratação e desnutrição, piorando a qualidade de vida desses pacientes, e por vezes evoluindo ao óbito ^(17,18)^.

Sabe-se que a EDSS é um instrumento que quantifica as incapacidades ocorridas durante a evolução da EM ao longo do tempo e estima-se que quanto maior o tempo de diagnóstico da doença, maior será a sua pontuação, assim como identificado na presente pesquisa ^(14)^. Compreende-se ainda, que a disfagia é mais comum em pacientes com quadros mais graves da doença, apesar dos escores da escala EDSS demonstrarem que alguns dos indivíduos dessa amostra apresentaram comprometimento leve na deglutição com escores da EDSS até quatro ^(19)^. Tarameshlu et al. identificaram em seu estudo que pacientes com disfagia apresentaram escores EDSS significativamente maiores do que em pacientes não disfágicos. Além disso, também observaram que a duração da doença foi significativamente maior no grupo de pacientes com disfagia ^(12)^. Por ser uma doença com progressão, pacientes com EM podem apresentar riscos ainda maiores para disfagia devido ao seu comprometimento motor e/ou sensitivo ^(12-20)^.

Os fármacos de primeira linha são utilizados em pacientes que apresentam EM no seu estágio inicial e possuem a doença menos ativa, ou seja, com menores incapacidades funcionais. Já as medicações de segunda e terceira linhas são usadas em indivíduos com progressão mais agressiva da doença, e que já falharam com a primeira estratégia terapêutica ^(9,19)^. Apesar de não haver subsídios suficientes para indicar que o tipo de fármaco tem influência sobre a disfagia, observou-se que a evolução da doença em relação ao tempo de diagnóstico e aos escores da escala EDSS superiores ao ponto de corte, mostraram relação com a alteração de deglutição.

Nesta amostra, foi significativo o número de pacientes com EM que referiram sintomas sugestivos de alterações de deglutição, considerando a autopercepção destes por meio da aplicação do questionário EAT-10. O mesmo indicou sofrer interferência nos seus resultados pelo tratamento e também pela forma de apresentação da doença, sugerindo que pacientes medicados com fármaco de primeira linha teriam menos queixas de deglutição, em virtude do estágio inicial da doença e, consequentemente, menor EDSS. Da mesma maneira que, indivíduos com EM do tipo surto-remissão apresentaram menos queixas do que os pacientes com EM primariamente progressiva.

Existem outros protocolos de autopercepção adotados para identificar disfagia em pacientes com doenças neurodegenerativas, em especial, o DYMUS para pessoas com Esclerose Múltipla. Porém, este é um instrumento que se limita a perguntas referentes ao ato de engolir, e não aos impactos sociais e psicológicos desses indivíduos causados por um transtorno de deglutição ^(13,16)^. O EAT-10 é um instrumento útil para detectar a existência de disfagia e monitorizar a resposta de um indivíduo ao tratamento ^(13)^. Este questionário mostrou-se útil como forma de triagem para a disfagia, pois quase metade dos pacientes com EM desta amostra relatou sinais sugestivos de distúrbios de deglutição como resultado do questionário, o que está associado ao nível de disfagia identificado pela GUSS. Em um estudo anterior, foi identificada a presença de disfagia orofaríngea em 90% dos pacientes avaliados com EM. A gravidade da disfagia foi relacionada com a forma clínica da doença, sendo constatado que a disfagia grave estava mais presente nas formas primariamente progressiva e secundariamente progressiva, e a disfagia leve e moderada foi mais encontrada na forma recorrente remitente ^(21)^. Dados estes que corroboram com os resultados da escala GUSS e EAT-10 deste estudo, os quais evidenciam que mais da metade dos indivíduos desta amostra apresenta disfagia orofaríngea de grau leve, bem como, EM do tipo surto-remissão.

O estudo apresentou limitações para afirmar sobre a interferência do tratamento proposto em relação ao risco de disfagia, devido à heterogeneidade dos grupos e dos diferentes comprometimentos motores dos pacientes evidenciados pela escala EDSS. Os demais resultados são condizentes com os achados da literatura, sendo possível afirmar que a autopercepção para o risco de disfagia dos indivíduos com EM demonstrada pelo EAT-10, se relaciona com os achados da avaliação clínica da deglutição feita através da GUSS. Sugere-se, ainda, futuras pesquisas com grupos homogêneos para resultados mais fidedignos em relação ao tipo de EM, a gravidade da disfagia e ao tratamento da EM e sua influência com os distúrbios de deglutição.

## CONCLUSÃO

Pode-se afirmar que houve associação entre os achados da avaliação clínica, do instrumento de autopercepção da deglutição e da escala de incapacidade motora em pacientes com EM.
